# Hemodynamic inefficiencies during stress hold key for comprehensive assessment after Fontan operation

**DOI:** 10.1016/j.xjtc.2023.11.003

**Published:** 2023-11-10

**Authors:** Joao F. Fernandes, Hannah Bellsham-Revell, James Wong, Caner Salih, Kuberan Pushparajah, Pablo Lamata, Adelaide de Vecchi

**Affiliations:** a School of Biomedical Engineering and Imaging Sciences, King's College London, London, United Kingdom; b Department of Congenital Heart Disease, Evelina London Children's Hospital, Guy's & St Thomas' Hospital, London, United Kingdom

**Figure:**
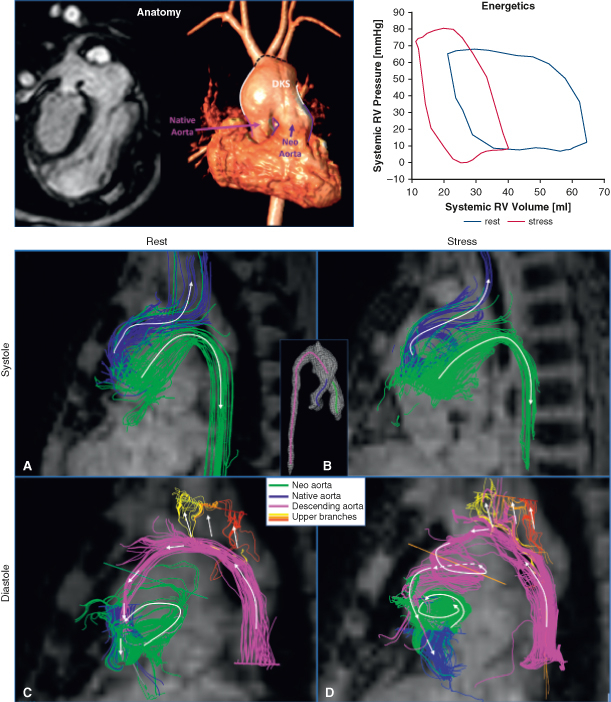
A-D, Flow distribution from 4D Flow MRI pathlines colored based on flow origin for rest (A and C) and stress (B and D) conditions during systole (A and B) and diastole (C and D)

**Central Message**

Post-Fontan arterial stiffness and flow hemodynamics are altered in stress, leading to preload restriction, 73% afterload increase, and severe restrictive retrograde flow toward myocardial perfusion.

In hypoplastic left heart syndrome and variants, Damus–Kaye–Stansel (DKS) anastomosis affects the shape of the reconstructed aorta and subsequently its conduit function and the myocardial workload. The DKS also provides vital retrograde flow toward the coronaries.^1^^,^^2^ We present a case of critical aortic stenosis with a borderline left ventricle (LV), where 4-dimensional (4D) flow analysis revealed new mechanistic insights into the relationship between DKS remodeling and flow.

## Case History

**Figure 1:**
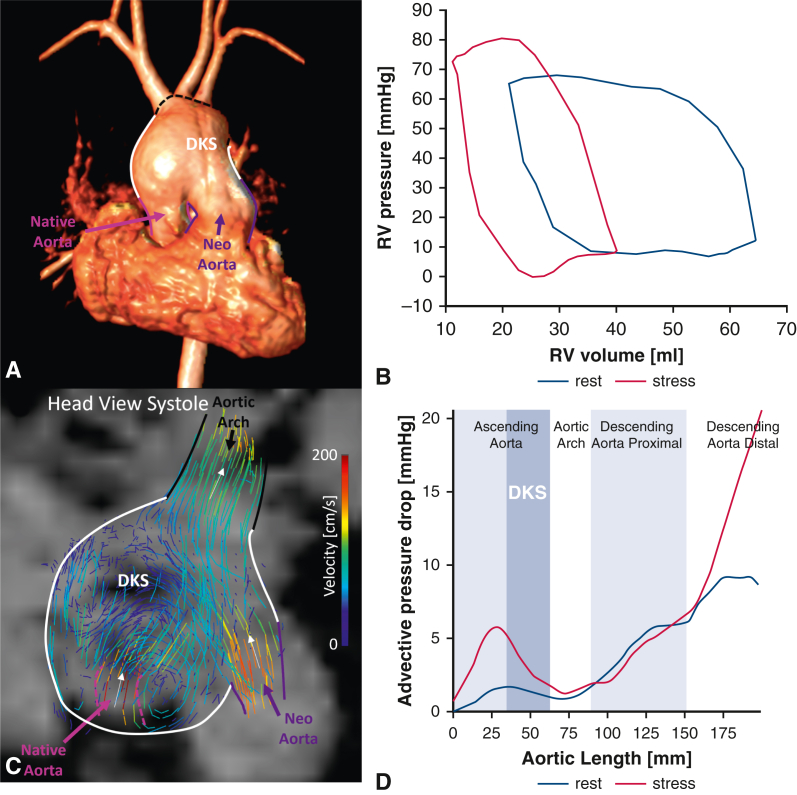
A, Three-dimensional MRI angiography of the DKS anastomosis. B, Pressure–volume loops of the systemic right ventricle at rest and under pharmacologic stress. C, Four-dimensional flow MRI streamlines showing the vortex in DKS region at peak systole. D, Comparison of conduit function at rest and stress, assessed by the advective pressure drop along the aortic length at peak systole.^3^*MRI*, Magnetic resonance imaging; *DKS*, Damus–Kaye–Stansel.

A 5.5-year-old boy with a systemic right ventricle (RV), a hypertrophied LV, endocardial fibroelastosis, and a dilated DKS underwent comprehensive post-Fontan operation follow-up for symptoms of effort intolerance following institutional ethical approval (21/LO/0650, September 28, 2021) (Figure 1). Informed consent for publication of study data was provided.

He had initially undergone balloon aortic valvuloplasty to resolve critical aortic stenosis followed by the hybrid procedure (bilateral pulmonary artery banding and ductal stent insertion). At posthybrid imaging, the LV contributed 25% of the cardiac output. Biventricular repair was, however, rejected due to restrictive LV physiology and the risk of the left heart becoming unable to support the systemic circulation without the development of pulmonary hypertension. Therefore, the patient underwent arch reconstruction and DKS with hemi-Fontan operation at 1 year, 4 months. An incision was made along the underside of the transverse arch and down the ascending aorta to the level of the transected main pulmonary artery. The distal arch and descending aorta were sutured together along their adjacent margins using continuous 6-0 PROLENE (Ethicon). An incision was made down the medial aspect of the descending aorta, and a patch of donor pulmonary homograft was used to augment the arch. A V-shaped cut in the main pulmonary artery was made adjacent to the native ascending aorta and the 2 vessels were sutured together. The patient then proceeded to fenestrated lateral tunnel Fontan completion at 3 years, 8 months.

Cardiac magnetic resonance imaging acquisition, including 4D flow, and simultaneous cardiac catheterization were performed post-Fontan operation during rest (fraction of inspired oxygen 0.3) and pharmacologic stress (fraction of inspired oxygen 0.3, dobutamine 10 μg/kg/min) under general anesthesia. The systemic RV showed adequate contractile reserve (end-systolic volume: 27 mL/m^2^ at rest vs 17 mL/m^2^ at stress) and inotropic response (end-systolic elastance: 1.22 vs 1.59) (Figure 1, *B*). Diastolic function improved during stress with the isovolumetric relaxation constant decreasing from 62 to 18 milliseconds. Despite this, preload fell (mean end-diastolic wall stress: 89 × 10^3^ dynes/cm^2^ vs 70 × 10^3^ dynes/cm^2^), suggesting limited pulmonary venous return.^3^ Ventricular afterload simultaneously increased (arterial elastance: 1.52 vs 2.63), signaling a deterioration of coupling between neoaorta and systemic RV (arterial elastance/end-systolic elastance: 1.25 vs 1.65).

**Figure 2:**
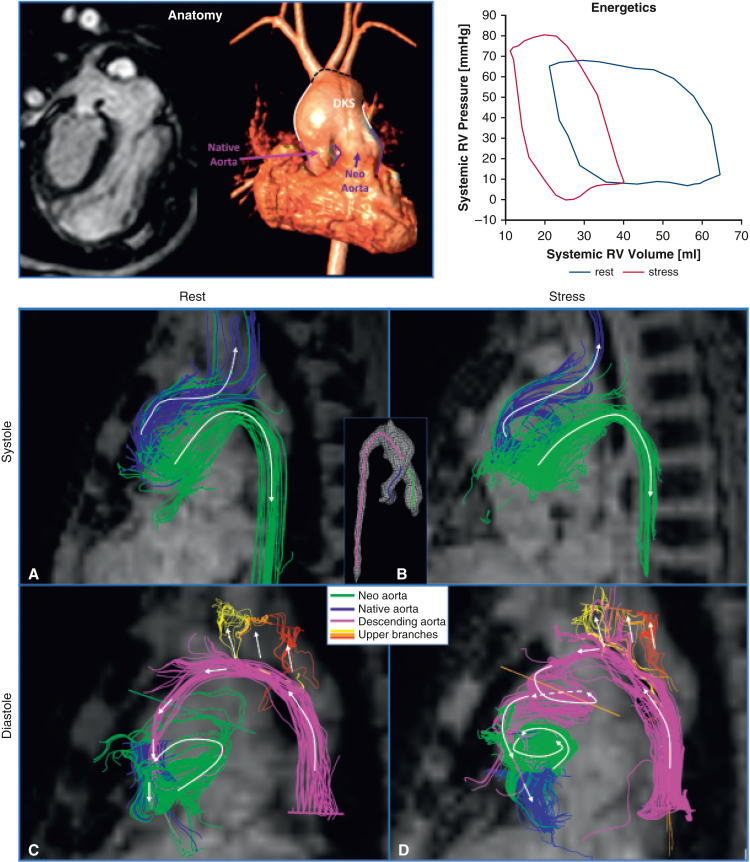
A-D, Flow distribution from 4D Flow MRI pathlines colored based on flow origin for rest (A and C) and stress (B and D) conditions during systole (A and B) and diastole (C and D). *MRI*, Magnetic resonance imaging.

**Figure 3:**
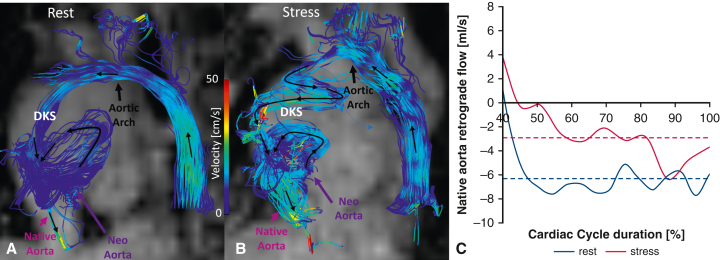
A and B, Four-dimensional flow MRI streamlines of retrograde flow from the descending aorta into the native aorta at rest and stress, respectively. C, Diastolic retrograde flow: consistently with the flow dynamics shown in (A) and (B), the retrograde flow rate is higher at rest than at stress, with *dashed lines* indicating mean flow rates (6.1 mL/s vs 3 mL/s). *MRI*, Magnetic resonance imaging.

To characterize this afterload noninvasively, aortic pressure gradients and energy inefficiencies were quantified from 4D flow magnetic resonance imaging (MRI) data.^4^ The greatest increases in systolic pressure gradients under stress occurred in the DKS (3.1 mm Hg) and descending aorta (7 mm Hg) (Figure 1, *D*). Pulse-wave velocity analysis showed a significant stiffness increase at the proximal descending aorta (61.1 Kpa at rest vs 163.6 Kpa at stress). At peak systole, vortical flow in the DKS caused a 2.5-fold raise in energy dissipation during stress (Figure 1, *C*). These inefficiencies cumulatively increased the afterload by 73%, as measured by *E*_*A*_. In systole, the native aortic flow fed the upper aortic branches, whereas the neoaortic flow was mostly channeled into the descending aorta (Figure 2, *A* and *B*) . In diastole, retrograde flow from the descending aorta perfused the upper branches before entering the native aorta with minimal vortical motion at rest (Figure 3, *A*). In contrast, during stress, it formed spiraling vortices with low velocity and delivered 53.8% less mean flow rate to the coronaries (Figure 3, *B* and *C*). Antegrade flow was also present in the neoaorta in early diastole in both conditions (Figure 2, *C* and *D*).

## Discussion

This case showed that the increase in afterload during stress caused aortoventricular uncoupling despite an adequate RV response. This functional burden was due to stiffness changes and flow inefficiencies, which potentially reinforced LV hypertrophy and DKS dilation.^5^^,^^E1^^,^^E2^

Interestingly, different aortic segments have adapted to perform complementary functions in systole and diastole. Although the native aorta fed the upper branches during systole, the descending aorta supported upper body perfusion in diastole. The diastolic flow to the coronaries was disrupted during stress, due partly to momentum dissipation in the dilated DKS, partly to the fact that, despite the RV's ability to relax, the aorta was unable to do so. This could be caused by the stiffer vascular tone recorded at stress without a significant increase in end-systolic pressure. This approach shows the value of 4D flow MRI to assess proxy metrics for identifying ventriculoarterial decoupling without the need for catheterization. Furthermore, the analysis of the stress datasets provides the first evidence of the sensitivity of coronary flow to the DKS remodeling, which is not apparent during rest. Although it is known that RV-dominant patients with hypertrophied LV commonly exhibit lower hyperemic myocardial blood flow,^E3^ no data exist on the role of the DKS remodeling in myocardial perfusion. Stress 4D flow MRI could thus be used to identify patients at risk of subclinical subendocardial ischemia despite good RV function and no anatomical obstruction, improving patient selection for treatment before myocardial damage occurs.

## Conflict of Interest Statement

The authors reported no conflicts of interest.

The *Journal* policy requires editors and reviewers to disclose conflicts of interest and to decline handling or reviewing manuscripts for which they may have a conflict of interest. The editors and reviewers of this article have no conflicts of interest.
